# Landmarks in pancreatic cancer studies

**DOI:** 10.1186/s12935-022-02803-8

**Published:** 2022-12-07

**Authors:** Fan Xu, Min Huang, Yun Bai, Xueshi Yin, Jingzhe Yan, Fangfang Liu, Jie Chen, Xiechuan Weng

**Affiliations:** 1 Department of Public Health, Chengdu Medical College, Chengdu, 610500 Sichuan China; 2 Department of Physiology, Chengdu Medical College, Chengdu, 610500 Sichuan China; 3 Department of Clinic Medicine, Chengdu Medical College, Chengdu, 610500 Sichuan China; 4 Department of Abdominal Oncosurgery-2, Jilin Province Tumor Hospital, Changchun, 130012 China; 5 Art college, Southwest Minzu University, Chengdu, 610041 Sichuan China; 6 Department of Orthopedics, Shanghai Institute of Traumatology and Orthopaedics, Ruijin Hospital, Shanghai Jiaotong University School of Medicine, Shanghai, 200025 China; 7 School of Chinese Medicine, Li Ka Shing Faculty of Medicine, The University of Hong Kong, Hong Kong, 999077 China; 8 Department of Neuroscience, Beijing Institute of Basic Medical Sciences, Beijing, 100850 China

**Keywords:** Landmarks, Pancreatic cancer

## Abstract

Pancreatic cancer is a rare but fatal disease. Patients present advanced disease due to the lack of or typical symptoms when the tumor is still localized. A high-quality image processing system has been in practice to detect the pancreatic tumor and determine the possibility of surgery, and preoperative methods, such as ERCP are increasingly used to complement the staging modality. Pancreaticoduodenectomy is one of the complicated surgeries with potential morbidity. The minimally invasive pancreatic resections, both robot-assisted and laparoscopic, have become a part of standard surgical practice worldwide over the last decade. Moreover, advancements in adjuvant chemotherapy have improved the long-term outcomes in current clinical practice. The systemic conservative treatment, including targeted agents, remains the mainstay of treatment for patients with advanced disease. An increasing number of studies are focused on modulating the pancreatic tumor microenvironment to improve the efficacy of the immunotherapeutic strategies. Herein, the role of preoperative therapy, the novel surgical strategy, and individualized systemic treatment in pancreatic cancer is investigated. Also, the randomized controlled studies that have defined the neoadjuvant and surgical management of pancreatic cancer have been summarized.

## Introduction

Pancreatic cancer is an intractable digestive system malignancy. Based on GLOBOCAN 2020 estimate [1], pancreatic cancer accounts for almost as many deaths (466,000) as cases (496,000). It is the seventh leading cause of cancer deaths in both sexes. A study of 28 European countries showed that because the rates of pancreatic cancer are stable relative to the declining rates of breast cancer, pancreatic cancer will surpass breast cancer as the third leading cause of cancer deaths by 2025 [2]. The survival rates for pancreatic cancer are extremely low, despite improvements in the overall 5-year survival from < 5% (1990s) to about 9% (2019) in the USA and Europe. The low survival rates could be partially attributed to the advanced stage at diagnosis in most cases, with only 20% of patients presenting early-stage, surgically resectable disease [3].

Data from China Pancreatic Disease Big Data Center showed that pancreatic cancer has three characteristics: low early diagnosis rate, low surgical resection rate, and low drug efficiency. In addition, it imposes a huge financial burden on the family and society. Cerullo et al. assessed the financial burden associated with the treatment options for resectable pancreatic cancer and reported that the median cumulative cost of gemcitabine with nab-paclitaxel was $74,051 (interquartile range: $38,929–$133,603) [4]. The cause of pancreatic cancer is complex and multifactorial, and an unhealthy lifestyle increases the incidence of the disease. Nonetheless, smoking remains a major cause of pancreatic cancer. Also, increased rates of diabetes and obesity may contribute to the high rates of pancreatic cancer [3]. Accumulating evidence suggested that heavy drinking increases the risk of pancreatic cancer [5, 6]. The genetic factors might explain 22–33% predisposition to the risk of pancreatic cancer risk [3].

This review outlines the current progress in pancreatic cancer in terms of the conservative treatment strategy, including immunotherapy and elucidates the immune cell modulation in tumor progression and surgical development for cancer treatment.

## Hallmarks of the histological and molecular characteristics

### Histological

Pancreatic tumors include cancers that arise from the endocrine or exocrine components of the pancreas with pancreatic adenocarcinoma. Most pancreatic cancers are pancreatic ductal adenocarcinomas (PDACs) (> 90%) [7]. PDAC is characterized by invasive, widely separated small tubular (ductal) structures embedded in fibroinflammatory (desmoplastic) stroma, which creates a scirrhous ill-defined lesion that renders difficulty in distinguishing PDAC from chronic pancreatitis both radiologically and pathologically. The infiltration pattern is characteristically subtle, which does not allow the formation of a well-defined mass; however, a highly insidious infiltration leads to peritoneal carcinomatosis with numerous small clusters, whereas the primary tumor may be small.

Nonetheless, it should be remembered that there is an array of other cancer types that occur in the pancreas. These cancers are classified by their cellular lineage: acinar cell carcinomas (acinar differentiation), neuroendocrine neoplasms (arising from the islets), solid-pseudopapillary neoplasms (showing no discernible cell lineage), and pancreatoblastomas (characterized by multiphenotypic differentiation, including acinar endocrine and ductal). Mesenchymal neoplasms, such as gastrointestinal stromal tumors and lymphomas, also occur in the pancreas [8].

In patients with hereditary germline and spontaneous somatic mutations, the pathogenesis of pancreatic cancer is well-defined in terms of precursor lesions that include pancreatic intraepithelial neoplasia (PanIN), intraductal papillary mucinous neoplasm (IPMN), and mucinous cystic neoplasm (MCN) [9].

### Molecular classification

Historically, pancreatic cancer was viewed as a single disease entity; however, it became clear that similar to other malignancies, such as breast cancer, it is molecularly diverse, and treatments are tailored to the biology of the tumor. The first landmark study to assess the global pancreatic cancer genome pattern was published in 2008, which included a genetic analysis of 24 patients with advanced pancreatic cancer and found that pancreatic cancer contained > 60 genetic changes, equivalent to disruptions in 12 core cell signaling pathways. The study confirms the genetic diversity of pancreatic cancer and lays the foundation for future research [10]. Advances in sequencing technology in recent years have greatly improved our understanding of pancreatic cancer at the molecular level.

**Fig. 1:**
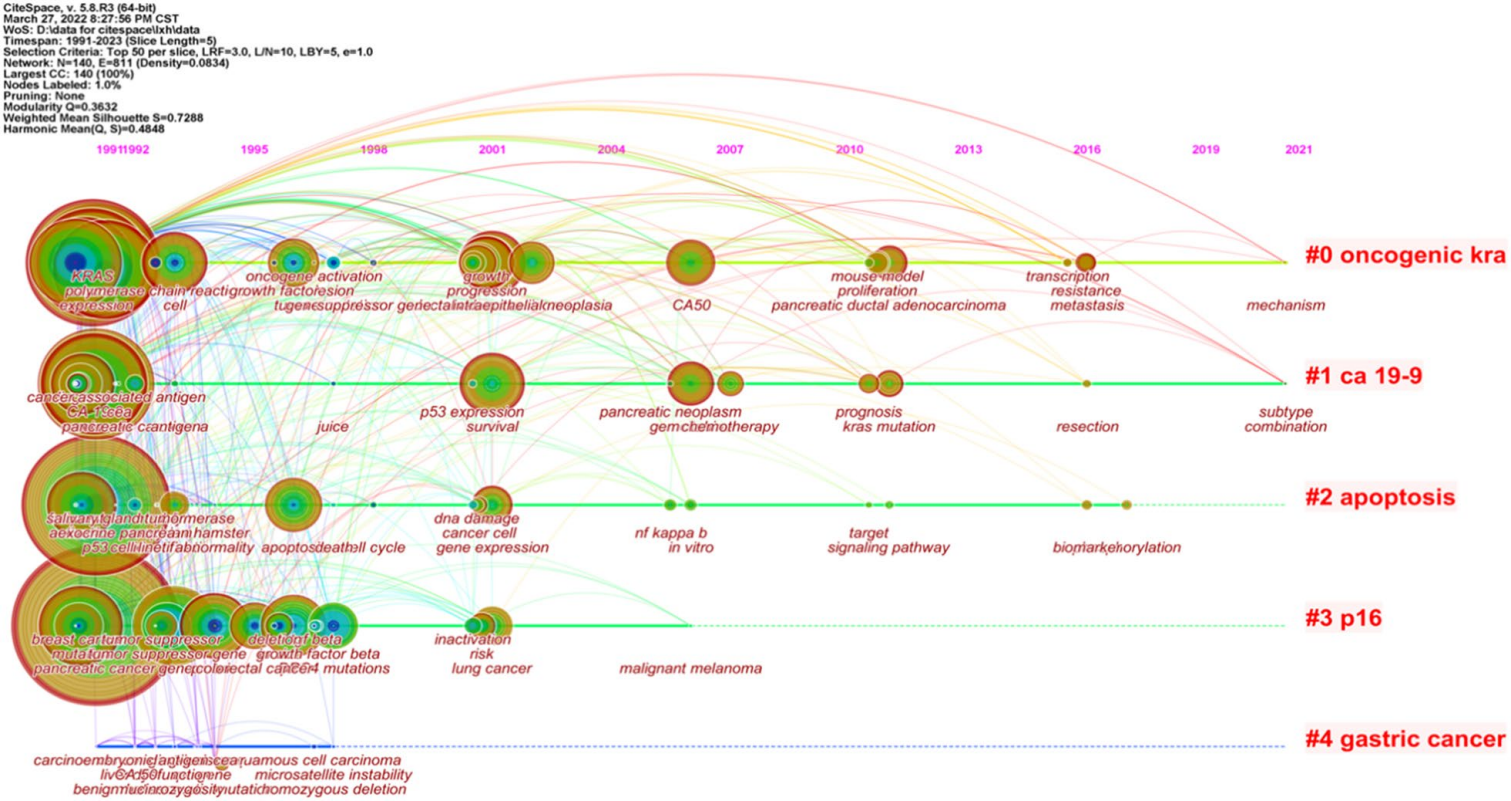
General genes in studies of pancreatic cancer

Collisson et al. [11] analyzed the transcriptional profiles of primary PDAC samples from several studies along with human and mouse PDAC cell lines and defined three subtypes, including classical, quasi-mesenchymal, and exocrine-like, according to specific gene expression. In addition, the study found that two genes associated with subtypes, GATA binding protein 6 (*GATA6*) and v-ki-ras2 kirsten rat sarcoma viral oncogene homolog (*KRAS*), implicated in both aspects of normal development and cancer pathophysiology (Fig. 1).

Pancreatic cancer is mesenchymal-rich, which makes capturing precise tumor-specific molecular information challenging [12]. Moffitt et al. have overcome this problem by applying blind source separation to diverse PDAC gene expression microarray data from primary, metastatic, and normal samples. A total of 50 genes related to the development of pancreatic cancer were screened based on tumor epithelial tissue and classified into two tumor-specific subtypes, including classical and basal-like, that have poor outcomes and are molecularly similar to basal tumors in bladder and breast cancers. Furthermore, 48 genes related to the development of PDAC were extracted from tumor stroma and defined as normal and activated stromal subtypes, which are independently prognostic.

Bailey et al. [13] performed whole-gene sequencing analysis on 456 pancreatic cancer samples. A total of 32 important cyclic mutation motifs and 10 key genetic signaling pathways were identified: *KRAS*, *TGF-β*, *WNT*, *NOTCH*, *ROBO/SLIT* signaling, G1/S transition, *SWI-SNF*, chromatin modification, DNA repair, and RNA processing. The expression analysis defined four subtypes, including squamous, pancreatic progenitor, immunogenic, and aberrantly differentiated endocrine-exocrine (ADEX). Squamous tumors are enriched in *TP53* and *KDM6A* mutations and have a poor prognosis. Pancreatic progenitor tumors expressed genes involved in early pancreatic development (*FOXA2/3*, *PDX1*, *and MNX1*). Immunogenic tumors consisted of upregulated immune networks, including acquired immune suppression pathways. ADEX tumors displayed upregulation of genes that regulate networks involved in *KRAS* activation, exocrine (*NR5A2 and RBPJL*), and endocrine differentiation (*NEUROD1 and NKX2-2*). The comprehensive evolution landmarks of genetic discovery in pancreatic cancer are displayed in Fig. 1.

The comparison of different genotypes revealed that 37/62 tumor cell genes analyzed by Collisson et al. and 32/50 tumor cell genes analyzed by Moffitt et al. comprised 707 tumor cell genes analyzed by Bailey et al. Furthermore, Collisson et al. identified 8 genes in tumor cells analyzed by Moffitt et al. Whether the genes were analyzed by Collisson et al. or Bailey et al., there was only a little overlap with the stromal genes analyzed by Moffitt et al. Despite a low gene overlap, all classifications were associated with pathological grade, a critical prognostic feature that reflects the intrinsic molecular characteristics of tumors.

#### Clinical staging

Accurate staging is the basis for guiding the diagnosis and treatment of malignant tumors and evaluating the prognosis, especially for pancreatic cancer, which is highly malignant and is challenging for diagnosis and treatment. The cancer staging system introduced by the American Joint Council on Cancer (AJCC) has become the gold standard for malignant tumor staging. Presently, clinical staging of pancreatic cancer is based on the eighth edition of the TNM staging system.

van Roessel et al. [14] reported that the eighth edition of the TNM staging system displays an equal distribution among stages and a modestly increased prognostic accuracy in patients with resected PDAC compared to the seventh edition. The revised T stage remains poorly associated with survival, whereas the revised N stage is highly prognostic. Taniuchi et al. [15] demonstrated that the combination of PODXL with ITGB1 and that of BCL7B with ITGB1 accurately predicted the postoperative outcomes of pancreatic cancer patients; these predictors were superior compared to the TNM staging system. The combination of PODXL with ITGB1 was rather beneficial as it was the most highly correlated with the postoperative outcomes.

## Screening and early diagnosis of pancreatic cancer

The United States Preventive Medicine Task Force (USPSTF) indicated that the potential benefits of screening for pancreatic cancer in asymptomatic adults do not outweigh the potential risks, and screening for pancreatic cancer in asymptomatic adults is not recommended. Since early screening is crucial to improve the overall prognosis of patients with pancreatic cancer, it should be performed in high-risk groups.

### Development of imaging technology

The diagnosis of pancreatic cancer depends on traditional imaging examination. With the rapid development of imaging technology and equipment, medical imaging, including transabdominal ultrasound (TAUS), computed tomography (CT), magnetic resonance imaging (MRI), and endoscopic ultrasonography (EUS), plays a critical role in the diagnosis of PDAC, which has different capabilities for the detection of early pancreatic cancer [16].

Diffusion MRI technology, including diffusion-weighted imaging (DWI) and intravoxel incoherent motion imaging (IVIM), has shown superior diagnostic efficacy [17]. Due to the limited diffusion of water molecules in pancreatic cancer focus, DWI sequences showed significantly high signal and significantly decreased diffusion coefficient and perfusion fraction of IVIM sequence, which can distinguish pancreatic cancer from other mass pancreatitis and autoimmune pancreatitis. Compared to CT, EUS can detect smaller solid lesions and has the added advantage of not using ionizing radiation, not requiring contrast agents, and obtaining cytopathological results sequentially. Especially, the endoscopic ultrasound-guided fine-needle aspiration (EUS-FNA) is the gold standard for the diagnosis of pancreatic cancer [18]. Additionally, radiomics is a new approach for image analysis, combined with artificial intelligence (AI) and computer-aided diagnosis system that facilitates radiographical diagnosis and step into the era of mass data and precision [19].

### Discovery of new serum marker

Serum cancer antigen 19 − 9 (CA 19 − 9) is the only marker approved by the United States Food and Drug Administration for use in the routine management of pancreatic cancer [20]. The low positive predictive value means that CA19-9 has no role in the mass screening of asymptomatic patients and is only appropriate for monitoring the response to treatment and as a marker of recurrent disease [21].

New serum markers combined with clinically common tumor marker detection improves the early diagnosis rate of pancreatic cancer. CA50 combined with tissue polypeptide antigen detection improves the detection rate of pancreatic cancer [22], and CA19-9 combined with CA125 detection significantly improves the diagnostic sensitivity of pancreatic cancer [23]. With the development of protein molecular technology and proteomics, several serum protein molecules characteristic of early pancreatic cancer have been identified, including matrix metalloproteinase MMP-2, MMP-9, [24] and serum galactoagglutinin-3 [25].

In recent years, the study on non-coding RNA (ncRNA) has developed rapidly and achieved a series of breakthrough results, which have established characteristic models with a strong diagnostic efficiency. In addition, the application of liquid biopsy involving circulating tumor cells (CTCs), circulating tumor DNA (ctDNA), and exosomes provides a new research direction for the early diagnosis of pancreatic cancer.

## Landmark of treatment

### Resectable pancreatic cancer

**Fig. 2:**
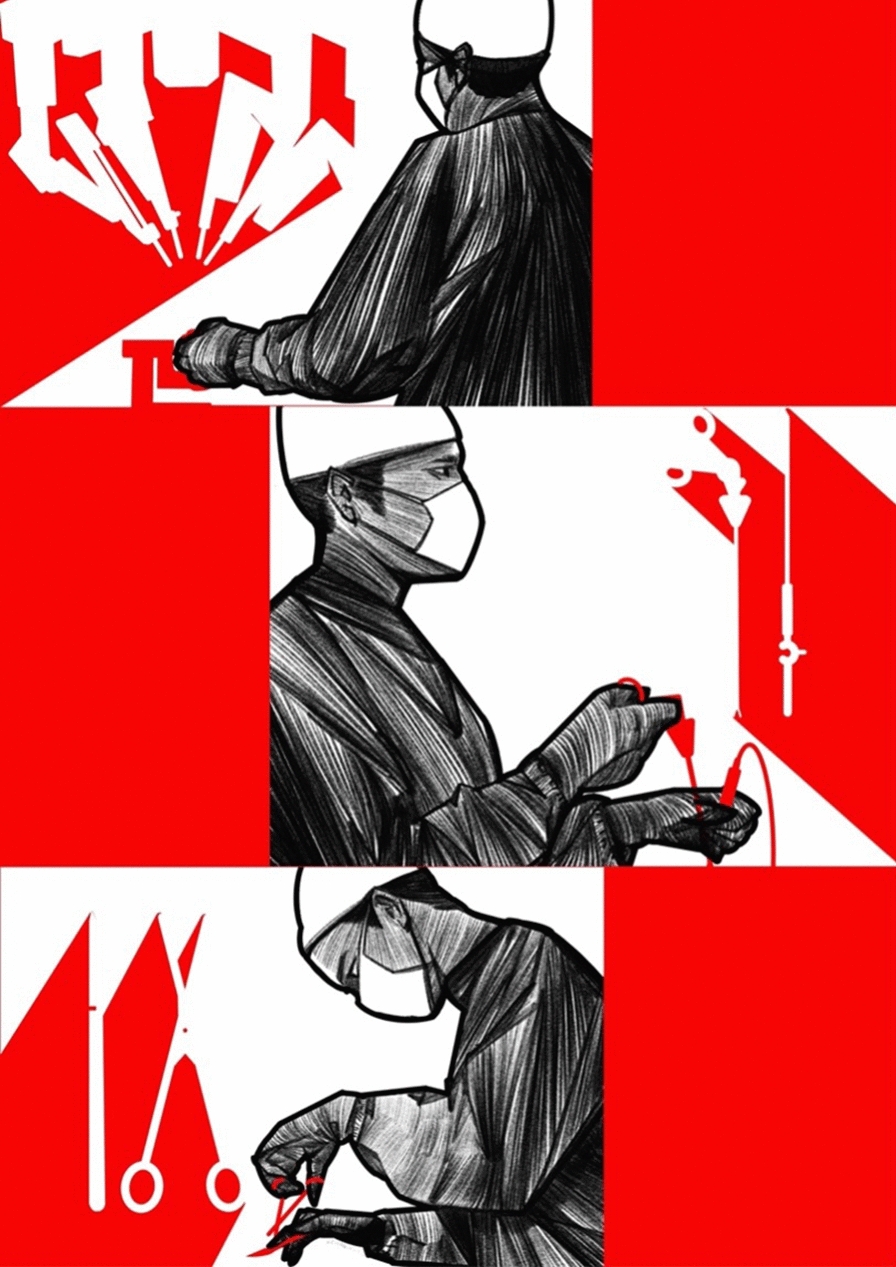
Landmarks in surgical operation pattern against pancreatic cancer (Drawn by FFL)

Pancreaticoduodenectomy (Whipple’s procedure), distal or total pancreatectomy, is the surgical option for the resection of pancreatic cancer. Trendelenberg performed a distal pancreatectomy to remove a tumor of the pancreas. Despite a poor postoperative outcome, this procedure marked the birth of pancreatic surgery [26]. In 1898, The first recorded attempt at a partial pancreaticoduodenectomy was by Alessandro Codivilla. After 21 days, the patient died of cachexia (2009). In 1909, Walter Kausch performed the first successful two-stage partial pancreaticoduodenectomy, and the patient survived for 9 months until dying of cholangitis, without evidence of visible tumor recurrence at autopsy [27]. About three decades later, Allen Whipple published his series of three patients with ampullary cancer, which marked the first report of a two-stage complete pancreaticoduodenectomy [28]. In 1942, Whipple reported the modification of this operation to a one-staged procedure and further modified it in 1946 [29]. The operative mortality of pancreaticoduodenectomy was > 30% at its inception and did not improve significantly. The median OS of patients with resectable pancreatic cancer was approximately 12 months in the 1960s, leading to questions about the curative intent of the procedure [30]. Thanks to advanced development in surgical robot, more and more patients in this disease benefit from the minor wounds, less infection and longer survival periods. The landmarks in surgical operation pattern against pancreatic cancer, from bottom to up, the complex manual operation to robot assistant operation. More details, the complex manual operation usually need more length wounds, see below part. Then thanks to the second generation technology celioscope, the area and length of wounds become smaller and smaller, see middle part. Recently, the AI and robot technology developing sharply, the robot assistant operation can save more time, conduct more complex operation, see above part, Fig. 2.

The Gastrointestinal Tumor Study Group (GITSG) trial heralded a new era of adjuvant therapy, and operative mortality declined to < 3%, as reported in the reviews in 1980s [30]. Subsequent clinical trials over the next several decades have pushed the boundaries of treatment of resectable pancreatic cancer, resulting in significant improvements in OS. The JASPAC 01 and PRODIGE-24 trials [31],[32] in the 2010s demonstrated significant improvements in median OS of resectable pancreatic cancer with adjuvant S-1 and FOLFIRINOX (a combination chemotherapy regimen consisting of oxaliplatin, irinotecan, fluorouracil, and leucovorin). In 2019, PREOPANC-1, the first phase III clinical trial of neoadjuvant therapy, showed benefits in disease-free survival, R0 removal, and decreasing pathologic lymph node rate, perineural infiltration, and venous infiltration as secondary endpoints [33]. However, a meta-analysis about the preoperative/neoadjuvant therapy in pancreatic cancer found that estimated median survival following resection was 23.3 (range 12–54) mouths for initially resectable tumors patients and 20.5 (range 9–62) mouths for and initially non-resectable tumors patients, and concluded that in patients with resectable tumor, survival after neoadjuvant therapy were similar to those of patients with primarily resected tumors and adjuvant therapy [34]. Therefore, neoadjuvant chemotherapy for the treatment of resectable pancreatic cancer remains controversial. According to the guidelines of the National Comprehensive Cancer Network (NCCN) and the Pancreatic Surgery Group of the Surgery Society of the Chinese Medical Association, the indications of neoadjuvant chemotherapy are: (1) Suspicious metastases are found in imaging examination; (2) Serum CA19-9 level increase significantly; (3) The primary tumor is large; (4) Regional lymph nodes are larger.

There is a growing interest in minimally invasive techniques for pancreatic surgery. Laparoscopic distal pancreatectomy was the first minimally invasive pancreatectomy. A meta-analysis demonstrated that laparoscopic and open distal pancreatectomy had comparable morbidity and mortality, with reduced blood loss and length of hospital stay in the minimally invasive group. Also, no difference in the positive rate of resection margins [35]. Further meta-analysis suggested that laparoscopic distal pancreatectomy is similar to open surgery, but the lack of primary evidence indicated that it could not be sup [36].

Interestingly, robotics has been applied to improve Whipple’s surgery. A meta-analysis of a retrospective cohort study found a lower incidence of complications and less margin involvement in the robotic group compared to open pancreatectomy [37]. However, these studies lack randomization, which makes them vulnerable to selection bias. Robotic surgery also requires significant capital investment; the cost-effectiveness assessments were not included in any of the articles.

### Borderline resectable pancreatic cancer and locally advanced unresectable pancreas

For borderline resectable and locally advanced unresectable pancreatic cancer, the 2020 version of the US NCCN guidelines has clearly defined neoadjuvant therapy as the clinical diagnosis and treatment standard that affirms the clinical application value of neoadjuvant therapy for such diseases and provides patients with the opportunity of surgical resection after receiving tumor transformation therapy. A single-arm phase II clinical trial investigated the effects of adjuvant chemoradiotherapy combined with FOLFIRINOX and the angiotensin II receptor antagonist losartan in patients with locally advanced unresectable pancreatic cancer. Consequently, this treatment plan provided a downstaging of locally advanced pancreatic ductal adenocarcinoma and was associated with an R0 resection rate of 61% [38]. Our previous systemic review and meta-analysis described the clinical efficacy of radiotherapy in neoadjuvant therapy for borderline resectable pancreatic cancer and local advanced unresectable pancreatic cancer; however, the treatment-related toxicity might significantly reduce the life quality of patients [39].

### Metastatic pancreatic cancer

The management of metastatic pancreatic cancer involves symptom control and management of jaundice, and gemcitabine is the therapeutic drug. A phase III randomized clinical study of 342 patients with untreated metastatic pancreatic cancer demonstrated that the median OS in the FOLFIRONOX group was 11.1 months compared to 6.8 months in the gemcitabine group. However, the incidence of adverse effects within the group receiving FOLFIRONOX was increased [40]. Another first-line phase III clinical study showed that the median progression-free survival was 5.5 months in the nab-paclitaxel-gemcitabine group (Gnp) compared to 3.7 months in the gemcitabine group; however, the rates of peripheral neuropathy and myelosuppression were increased [41]. FOLFIRINOX and Gnp are options for the treatment of patients with metastatic pancreatic cancer and a satisfactory performance status.

### Other treatment strategies

Pancreatic cancer has unique characteristics, including dense stroma and tumor microenvironment filled with immunosuppressive intermediates, which form a solid barrier against pancreatic cancer immune cell and drug infiltrations. Immunotherapy is active against melanoma, kidney cancer, non-small cell lung cancer, and other malignant tumors. Presently, the immunotherapy effect of pancreatic cancer is not optimal, but as more immune mechanisms are being revealed and clinical studies are underway, significant progress is expected in the future.

With the understanding of the mechanism of pancreatic cancer, additional targets for PDAC therapy are being discovered. The POLO research on gBRCAm [42], phase I and II clinical trials on epidermal growth factor receptor (EGFR) target drugs, and the study on PARP-1/2 inhibitors [43] are exploring the value of these drugs in the treatment of pancreatic cancer.

## Conclusion

Pancreatic cancer is a devastating malignancy disease with a restricted approach to treatment. Thus, improving OS and treatment outcomes in the patient will rely on multidisciplinary cooperation in imaging, surgical procedures, radiation, and personalized therapies. Since the clinical progress is gradual, our insight into the molecular biology of PDAC and the tumor and inflammatory microenvironment needs further exploration.

The tumor and inflammatory microenvironment is characterized by an abundance of immunosuppressive cells and a highly fibrotic stroma that prevents infiltration of immune effector cells. The ablative techniques have the potential to overcome these factors. It has been hypothesized that ablation induces anti-tumor immune responses by increasing the availability of tumor-specific neoantigens in an inflammatory context. Numerous preclinical studies demonstrated that radiation therapy, thermal ablation, and IRE induce systemic anti-tumor immune responses in multiple tumor types. The current data showed improved OS in the postoperative therapies after surgery. Nonetheless, monitoring the tumor response to postoperative treatment is challenging. Thus, it is essential to improve the sensitivity of pancreatic cancer to immunotherapy and improve the outcomes.

We are stepping towards an exciting era where a better understanding of tumor biology, novel therapeutic targets, and innovative clinical trial designs and protocols will fetch data to illuminate the treatment and termination of pancreatic cancer.

## Data Availability

The original contributions presented in the study are included in the article/supplementary material. Further inquiries can be directed to the corresponding authors.
